# To Waste or Not to Waste: Questioning Potential Health Risks of Micro- and Nanoplastics with a Focus on Their Ingestion and Potential Carcinogenicity

**DOI:** 10.1007/s12403-022-00470-8

**Published:** 2022-03-22

**Authors:** Elisabeth S. Gruber, Vanessa Stadlbauer, Verena Pichler, Katharina Resch-Fauster, Andrea Todorovic, Thomas C. Meisel, Sibylle Trawoeger, Oldamur Hollóczki, Suzanne D. Turner, Wolfgang Wadsak, A. Dick Vethaak, Lukas Kenner

**Affiliations:** 1 Division of Visceral Surgery, Department of General Surgery, Medical University of Vienna, Vienna, Austria; 2 Department of Internal Medicine, Division of Gastroenterology and Hepatology, Medical University of Graz, Graz, Austria; 3 Center for Biomarker Research in Medicine (CBmed), Graz, Austria; 4 Department of Pharmaceutical Sciences, Division of Pharmaceutical Chemistry, University of Vienna, Vienna, Austria; 5 Materials Science and Testing of Polymers, Montanuniversitaet Leoben, Styria, Austria; 6 General and Analytical Chemistry, Montanuniversitaet Leoben, Styria, Austria; 7 Division of Systematic Theology and its Didactics, Faculty of Catholic Theology, University of Wuerzburg, Wuerzburg, Germany; 8 Mulliken Center for Theoretical Chemistry, University of Bonn, Bonn, Germany; 9 Department of Pathology, University of Cambridge, Cambridge, CB2 1QP UK; 10 Central European Institute of Technology, Masaryk University, 602 00 Brno, Czech Republic; 11 Division of Nuclear Medicine, Department of Biomedical Imaging and Image-Guided Therapy, Medical University of Vienna, Vienna, Austria; 12 Department of Environment and Health, Vrije Universiteit Amsterdam, Amsterdam, Netherlands; 13 Unit of Marine and Coastal Systems, Deltares, P.O. Box 177, 2600 MH Delft, Netherlands; 14 Christian Doppler Laboratory for Applied Metabolomics, Medical University of Vienna, Vienna, Austria; 15 Division of Experimental and Laboratory Animal Pathology, Department of Pathology Medical, University of Vienna, Vienna, Austria; 16 Unit of Laboratory Animal Pathology, University of Veterinary Medicine Vienna, Vienna, Austria

**Keywords:** Microplastic, Nanoplastic, Carcinogenesis, Human health, Bioethics issue

## Abstract

Micro- and nanoplastics (MNPs) are recognized as emerging contaminants, especially in food, with unknown health significance. MNPs passing through the gastrointestinal tract have been brought in context with disruption of the gut microbiome. Several molecular mechanisms have been described to facilitate tissue uptake of MNPs, which then are involved in local inflammatory and immune responses. Furthermore, MNPs can act as potential transporters (“vectors”) of contaminants and as chemosensitizers for toxic substances (“Trojan Horse effect”). In this review, we summarize current multidisciplinary knowledge of ingested MNPs and their potential adverse health effects. We discuss new insights into analytical and molecular modeling tools to help us better understand the local deposition and uptake of MNPs that might drive carcinogenic signaling. We present bioethical insights to basically re-consider the “culture of consumerism.” Finally, we map out prominent research questions in accordance with the Sustainable Development Goals of the United Nations.

## Back to the Roots: Origin, Release and Uptake of Plastic Particles

Back in the 1950s, when plastic revolutionized the market, nobody anticipated the other side of the coin, until recently. Over the last few years, several studies have highlighted massively accumulating volumes of small plastic debris and its evident hazardous environmental consequences, calling for further in-depth investigations on the effect of micro- and nanoplastics (MNPs) on human health in general and on carcinogenesis in particular.

The consumption of plastic particles, commonly termed microplastics (MPs; 0.1–5000 µm in size) and nanoplastics (NPs; < 0.1 µm in size) can occur either directly through the food chain or indirectly via the ingestion of inhaled and regurgitated particles (Wright and Kelly 2017). According to a study by the Marine Biology and Ecology Research Centre (MBERC) in England, the plastic load released from clothes made of synthetic fibers (polyester, polyester-cotton and acrylic) amounts to over 700,000 large MP fibers per machine wash (per 6 kg load) (Napper and Thompson 2016) that end up in waste water. Tons of plastic particles reach their final destination in the sea to enter the food chain through ingestion by marine life (Cho et al. 2019; Van Cauwenberghe and Janssen 2014), through sea salt (Karami et al. 2017; Kosuth et al. 2018; Yang et al. 2015) and/or drinking water (Mason et al. 2018; Schymanski et al. 2018) to further reincarnate on our dining tables. Recent studies have also indicated the presence of MPs in some terrestrial food items, such as edible fruit and vegetables and store-bought rice, but further research is needed to replicate these findings (Dessì et al. 2021; Oliveri Conti et al. 2020). Translated into more imaginable numbers, on average we ingest five grams of MPs per week per person (roughly corresponding to the mass of a credit card) depending on the region in which we live, our lifestyle, and diet (Senathirajah and Palanisami 2021). However, in vitro human cell and in vivo mammalian models suggest that only a limited fraction of the smaller plastics particles will be absorbed by the human body (reviewed by Wright et al. (Wright and Kelly 2017)). A study of human consumption of MPs estimated the ingestion of 90,000 particles through recommended levels of water intake annually from bottled sources of water, compared to 40,000 MPs through tap water only (Cox et al. 2019). The incidental annual human ingestion of plastic particles in the form of airborne MP fibers during an evening meal has been estimated to range between 13,731 and 68,415 fibers per person (Catarino et al. 2018). As a result, MPs have been detected in feces of different species including humans (Pérez-Guevara et al. 2021; Schwabl et al. 2019; Zhang et al. 2021). A study group from Germany demonstrated widespread contamination of mineral water with xenohormones leaching from plastic bottles (polyethylene terephthalate (PET)) that possessed potent estrogenic activity in vivo (Wagner and Oehlmann 2009) which can have carcinogenic activity in the body of the consumer (Acconcia et al. 2017). Furthermore, in a biomonitoring study conducted by the U.S. Center of Disease Control (CDC), thirteen different phthalate metabolites (i.e., PET) were detected in the urine of 2636 participants (CDC 2019). Placental uptake of twelve different types of MPs (between 5 and 10 µm), with polypropylene (PP) among others, was detected in four of six human samples (Ragusa et al. 2021); another study demonstrated the uptake of fluorescent polystyrene (PS) beads with a diameter up to 240 nm in an ex vivo human placental perfusion model (Wick et al. 2010). Recently, a dataset was published concerning plastic particle release from infant feeding bottles, demonstrating values ranging from 14,600 to 4,500,000 MPs (> 1 µm) ingested per capita per day (Li et al. 2020b). Unfortunately, sterilization of baby milk exacerbates the issue whereby the process of mixing milk powder with hot water at a minimum of 70 °C, shaking and cooling down to feeding temperature, induces thermal and mechanical stress to the bottle material that might further aggravate MNP release (WHO/FAO 2007).

The fact that humans are such a powerful geological force led to the designation of the Anthropocene or “Age of Man” as a new geological epoch, popularized by Paul J. Cruzen, Nobel Laureate in Chemistry in 2002 (WHO/FAO 2007). As a cultural concept, it has the capacity to challenge established narratives and emphasizes the eradication of established knowledge boundaries and the stimulation of collaboration between different disciplines. Humanity has already introduced a massive amount of plastic into the atmospheric, terrestrial, and aquatic environments, making plastic waste so ubiquitous that it will even contribute to an identifiable fossil trail for generations to come. In addition to impacting entire ecosystems (Andrady 2015), it could also have an as yet completely unexplored impact on human health.

## The Good, the Bad, and the Ugly of Plastics

Plastics are synthetic or semi-synthetic organic materials of a high molecular weight, usually produced from mineral oil through highly efficient energetic and economical procedures. Plastic molecular chains mainly consist of carbon, hydrogen, oxygen, nitrogen, chlorine, fluorine, and silicon atoms. Plastics are easy to mold into complex shapes and forms, are extremely durable, lightweight, corrosion-resistant, thermally and electrically insulating, and offer a wide mechanical and multifunctional performance range. Their versatility and cost-effectiveness led to a spectacular exponential increase in annual global plastics production from 1.7 million tons in 1950 to 359 million tons in 2018. Asia is the major producer (51%) followed by the countries of the North American Free Trade Agreement (NAFTA) (18%) and Europe (17%) (PlasticsEurope 2019). Major types of commercial plastics reported in the literature as contributing to MNP prevalence include polyethylene (PE), PP, polyvinyl chloride (PVC), polyurethanes (PUR), PET, PS, Polymethyl methacrylate (PMMA), styrene-butadiene rubber (SBR), and polyamide 66. The basic characteristics, fields of application, and market share of these polymer types are given in Table 1. In addition to synthetic polymers or mixtures thereof, commercial plastics are typically mixed with additives and fillers/reinforcement to further enhance performance properties and include different antioxidants and stabilizers, plasticizers (bisphenol-A (BPA) and phthalates), flame retardants, colorants as well as various inorganic particles (sizes ranging from nanometer to millimeter), and organic and inorganic fibers (Ambrogi et al. 2017). In addition, residues of the monomers of the starting material are typically still prevalent in the plastics (Fries et al. 2013). Today, this plastic material is irreplaceable in various technologies and applications, and also plays an important role in terms of resource efficiency. Prominent and common plastic resource savers include building insulations (i.e., reducing heating demand), lightweight vehicle components (i.e., dampening of fuel consumption), and packaging (i.e., extended storage life of foodstuffs). Plastics are also essential for regenerative energy production (e.g., solar-thermal and solar-electrical devices, wind turbines) (Andrady and Neal 2009; Bonten and von Weizsäcker 2014). In healthcare, plastics are viewed as being irreplaceable and facilitate higher hygienic standards (e.g., protheses, examination gloves, sterile syringes, adhesive bandage strips, blood bags and tubes, heart valves). In terms of overall ecological characteristics (i.e., energy demand, emissions, and recyclability), plastics are superior to other materials in numerous applications.

**Table 1:** Characteristics, fields of application, and market share of the major types of commercial plastics

Plastic type	Chemical structure (Ehrenstein 2011)	Morphology	Density [g/cm^3^]	Important characteristics	Fields of application	Demand distribution [%] 2018
Polyethylene (PE)		Semi-crystalline	0.91–0.96	Medium stiffness and strength (mechanically flexible), chemical-resistant, considered food safe, weldable, cost-efficient	Bags, agricultural films, food packaging, bottles, houseware, toys, pipes	29.7
Polypropylene (PP)		Semi-crystalline	0.90–0.91	Moderately stiff and dimensionally stable, chemical-resistant, considered food safe, cost-efficient	Food packaging, wrappers, containers, pipes, automotive parts	19.3
Polyvinyl chloride (PVC)		Amorphous	1.16–1.55	Soft to rigid (dependent on plasticizer concentration), dimensionally stable, chemical-resistant, weather-resistant, flame-retardant, cost-efficient	window frames, profiles, pipes, cable insulation, hoses, pools	10.0
Polyurethane (PUR)		Amorphous -cross-linked	0.01–1.25	Wide range of properties dependent on applied monomers (see applications)	Cleaning products, furniture, sealants, insulation, synthetic leather, varnish, adhesives	7.9
Polyethylene terephthalate (PET)		Semi-crystalline	1.38–1.40	Rigid, dimensionally stable, chemical-resistant, considered food safe, high transparency	Bottles for drinks and cleaners, textile fibers	7.7
Polystyrene (PS)		Amorphous	0.02–1.05	Rigid, brittle, dimensionally stable, susceptible to stress corrosion cracking, partly chemical-resistant, considered partly food safe, cost-efficient	Food packaging, building insulation, electronics, leisure	6.4
Styrene-butadiene rubber (SBR) (Yuen and De Snaijer 2016)		Amorphous—cross-linked	1,3–1,6	Flexible, high tensile and impact strength, resilience, abrasion resistance, chemical resistance, cost-efficient	Car and light truck tires, insulation for wire and cabling, gaskets, adhesives, shoe soles, coated fabrics, chewing gums	–
Polymethyl methacrylate (PMMA) (La Mantia 2002)		Amorphous	1,14–1,21	Crystal clear transparency to light, weatherproof, chemical resistance	Windows, aircraft canopies, lighting fixtures	–
Polyamide 66 (Nylon 66) (Intratec Nylon 66 Production Cost Reports 2019)		Semi-crystalline	1,14	Hardness, abrasion resistance, wear resistance, chemical resistance	Fibers for textiles and carpets, fishing lines, food packaging	–

As for degradation of plastics, various environmental processes occur through complex pathways involving hydrolysis, mechanical abrasion, thermal degradation, photodegradation, and biodegradation (of note, biodegradation of most MNPs, excluding exclusive biodegradable types of plastics, is hampered by a lack of heteroatom scaffolds) (Andrady 2015). These degradation processes are generally very slow, decreasing the size of plastic particles in the range of 0–10^3^ µm/year, depending on the plastic type (Min et al. 2020) and the environmental conditions (Chamas et al. 2020), producing particles that vary in size (Lambert and Wagner 2016) and shape (ter Halle et al. 2016). Furthermore, photodegradation of plastics alters the chemical composition of these materials, introducing highly polar carbonyl groups. A recent study by Rowenczyk et al. showed that oxidation occurs at up to 600 µm depths from the surface of plastic objects (Rowenczyk et al. 2020). Thus, the surface of plastics in the environment contains oxidized, less hydrophobic moieties in varying amounts, which facilitates the adsorption of environmental compounds (Prunier et al. 2019; Rowenczyk et al. 2020). Consequently, a plethora of different compounds can be produced from plastic waste with highly diverse structures, which is one of the major challenges in the characterization of their impact on health (EFSA 2016).

While primary MNPs are present in the environment by direct means (accounting for 15–31% of ocean debris), e.g., from textiles during washing (35%), tire wear (28%), and cosmetics (2%), secondary MNPs arise following the fragmentation of plastic items, polymeric coatings, and/or plastic debris, e.g., plastic bottles, bags, and fishing nets, or by abrasion or aging-induced embrittlement (69–81% of ocean debris) (Nature 2019). The resulting small plastic debris detected in water, soil, air, and food is heterogeneous in nature with a large variety of sizes and shapes (predominantly fibers but also particles, fragments, and films) and has a highly complex composition, including polymeric materials and mixtures of chemicals (residual monomers, additives, and adsorbed chemical contaminants), biomolecules, and microorganisms (Barboza et al. 2018; Vethaak and Leslie 2016; Vethaak and Martínez-Gómez 2020).

The plastic additives or the chemical contaminants that become bound to MNPs in the environment (e.g., hydrophobic organic contaminants, heavy metals) can have a variety of toxic effects, including potential carcinogenic and epigenotoxic effects. BPA, for example, is widely used in the production of plastics and synthetic resins. It causes a wide range of disruptive effects in the body, partly because it interferes, at very low doses, with the function of various hormones. Phthalates and some of the brominated flame retardants have been shown to have similar adverse effects (Groh et al. 2019; Lithner et al. 2011). These endocrine disrupting chemicals (EDCs) can alter fetal programming at an epigenetic level, which can be passed down through generations and may play a role in the development of various chronic disorders later in life, such as metabolic, reproductive, and degenerative diseases, as well as some forms of cancer (Martínez-Ibarra et al. 2021). MPs can also release carcinogenic monomers, such as propylene oxide and vinyl chloride (Lithner et al. 2011). These substances are either left behind during the production process or are released as the plastics breakdown. Yet, there is controversy about the extent to which these substances are released from plastic products (EFSA 2016).

Plastic additives and other associated chemicals in plastic that leach out over the product lifecycle are generally ubiquitous global contaminants, exposing humans even before MNP ingestion occurs. Both absorbed chemical toxicants and additives in ingested MNPs often do not contribute significantly to the observed total chemical body load from all exposure routes (ingestion, inhalation, and dermal absorption), as chemical partitioning models predict (Koelmans et al. 2016). Moreover, absorption by MPs can also have a beneficial effect, as chemicals are excreted more easily in this manner, via the feces. In contrast, data are available for chemical contaminants of organic as well as plastics of an inorganic nature—most of them are persistent, bio-accumulative, and/or toxic—that adhere to MNPs (Mato et al. 2001) and might facilitate their toxicity or uptake by different organisms. Examples of contaminants are phthalates (Fossi et al. 2012), persistent organic pollutants (POPs), polychlorinated biphenyls (PCBs) and polybrominated diphenyl ethers (Bouwmeester et al. 2015; Ogata et al. 2009), polycyclic aromatic hydrocarbons (PAHs), organochlorine pesticides, (Bouwmeester et al. 2015), and cations of partially toxic ions of heavy metal elements such as aluminum, chromium, manganese, iron, cobalt, nickel, zinc, cadmium, and lead (Holmes et al. 2012; Rochman et al. 2014). However, the potential combined implications of MNPs and associated chemical contaminants on human health are not well understood and remain to be elucidated (Campanale et al. 2020). Especially, NPs and small MPs carrying chemical substances as they are able to cross cell membranes and may enhance the bioavailability of the chemical, analogous to nanosized polymeric drug delivery vehicles and thus deserve special attention. Moreover, it was reported that microorganisms, such as plastic decomposing organisms and opportunistic human pathogens accumulate on plastic debris (Harrison et al. 2011, 2014; McCormick et al. 2014; Zettler et al. 2013). The World Health Organization (WHO) considers the risks from pathogens in MP-associated biofilms to be far lower than the risk posed by the high concentrations and diversity of pathogens present in human and livestock waste (Organization 2019). Clearly, to date the consequences and impact of plastic debris and decay products for ecosystems and to human health have not been systematically and intensively investigated. In addition to MNPs, there is evidence that other contaminating particles, such as titanium dioxide (TiO_2_) and aluminosilicates (such as kaolinite) might be engulfed in gut tissue and affect human health (Powell et al. 1996a, b, 2000, 2007), but are not covered in these particles/chemicals due to their inorganic origin and different physico-chemical properties.

## “We Shall Require a Substantially New Manner of Thinking if Mankind is to Survive” (Einstein 1949)

An in-depth analysis of the novel class that is MNPs is particularly necessary in the age of the Anthropocene. As mentioned above, Paul J. Crutzen indicated that humans are the most important factor influencing atmospheric, aquatic, and terrestrial processes on Earth. In February 2000, at the annual meeting of the International Geosphere-Biosphere Programme, Crutzen brought the term Anthropocene to the attention of participants when he argued that we should no longer speak of the Holocene. Until then, the Holocene, which was placed at the end of the Pleistocene about 12,000 years ago, was the primary term used by experts to refer to Earth's age. Soon after, alternative concepts to the Anthropocene developed (Horn and Bergthaller 2019). The science theorist Donna Haraway coined the term Chthulucene instead, to illustrate that humans are not masters of their activities. Thus, Chthulucene clarifies that humans are deeply interconnected with other living beings and the environment (Haraway 2016). Between the proposed epochs Anthropocene and Chthulucene, it is important to conduct studies on MNPs. On the one hand, humans do not reduce plastic production and consumption by denouncing the problems of MNPs (Anthropocene). On the other hand, MNPs already act as “agents” (Latour 1999) and affect our environment and health in ways we are far from understanding and consequently that we cannot control (Chthulucene). Studies of the effects of MNPs on (the environment and) human health will allow us to better understand and possibly narrow down their negative impact on humans and the environment. Moreover, the possible catastrophic consequences demand that we must develop a different approach to plastic, and generally propagate and practice a different lifestyle, first and foremost re-considering the “culture of consumerism” (McFague 2013). As mentioned before, sound studies on the impact of MNPs on the environment are available, also from a cultural studies perspective, analyzing MNPs under conditions of the Chthulucene (Bergman 2019). We also want to elaborate on the impact of MNPs on humans as an interconnected species and discussion of recent findings will contribute to the sustainability debate. Humans are deeply interconnected with other (living) things and the environment. Physicist and philosopher Karen Barad has developed the term “intra-action” to illustrate that the autonomous subject does not exist. Humans and their health depend on other (living) things, including the waste originally produced by humans, which in turn “intra-acts” with humans (Barad 2007). A look back at science underlines the importance of tracing the intra-action of MNPs with humans: In 1891, the Russian chemist Alexander Dianin first synthesized BPA (Dianin 1891). Afterward, British researchers discovered its estrogenic effect and already used it in a therapeutic context as an estrogenic agent (Dodds and Lawson 1936). BPA is still a component of many plastic products nowadays.

## Ingestion and Deposition of Plastics in the Gastrointestinal Tract

The lack of consistency and standardization of sampling and analytical methods for detection of MNP pollution inhibits a global comparison of MNP deposition (Van Cauwenberghe et al. 2015). Geographical variations in MNP pollution are not only influenced by anthropogenic factors, but also by environmental causes such as oceanic currents, wind direction, and atmospheric deposition, driving the distribution of MNP particles on the planet (Barletta et al. 2019). To get an idea of regional differences in MNP exposure for humans, a comparison of the MNP load in filter feeders like mussels can be used as a surrogate. For example, in China 0.9–4.6 MP/g were found in mussels, whereas in Europe the range detected is from 0 to 0.51 MP/g (De Witte et al. 2014; Li et al. 2016). According to a conservative example presented by the European Food Safety Authority (EFSA), the consumption of 225 g mussels results in an exposure to 7 µg of plastics (assuming an estimated weight of 25 µg and a density of 0.92 g/cm^3^). Since mussels are eaten without removing the digestive tract where the maximum MP load is located, they are the most worrisome form of seafood with the highest known load of MPs (EFSA 2016). The gastrointestinal tract is the organ most exposed to plastic particles, since it has recently been shown that between 106 and 142 MP/day are ingested with food, 174–349 MP/day via bottled water and overall, per week up to 5 g MP are ingested (Cox et al. 2019; Senathirajah et al. 2021). However, no toxicology data are available concerning the effects of MNPs on human health and their risks for, and potential roles in cancer development.

To consider a potential pathogenic role in humans, one should consider the routes of exposure and the cells with which MNP may interact. On ingestion in the diet, MNPs move through the gastrointestinal tract where they have been shown to interact with the microbiome. MNPs in the gastrointestinal tract have been shown to be degraded by microbes (and fungus) (Yuan et al. 2020), while plastic particles themselves induce changes to the composition of the gut microbiome (recently reviewed by Fackelmann et al. (2019)). For example, MNP exposure in the diet was associated with a decrease in the diversity of the gut microbiome as well as taxonomic changes in mice. In the same study, increased intestinal permeability and changes in amino acid and bile acid metabolism, and hepatic lipid metabolism (Jin et al. 2019; Lu et al. 2018) were shown. Interestingly, the effects of MNP on the mammalian gut microbiome, including changes in microbiome diversity, an increase in potentially pathogenic bacteria, a decrease in commensal gut bacteria, and resulting metabolic dysfunction, resemble common findings in chronic human diseases such as diabetes, obesity, or chronic liver disease (Weiss and Hennet 2017).

It remains to be elucidated how MNP particles alter the microbiome although one might speculate that they may directly affect bacterial growth and/or metabolism by their physical presence, that they carry EDCs or are themselves polluted with microbial communities that alter the gut microbiome. The latter has been shown for marine microbiomes and zebrafish (Wan et al. 2019) but needs to be investigated in humans (Rosato et al. 2018). The effects of additives on the human gut microbiome are also poorly understood. The widely used plasticizer diethyl-hexyl phthalate (DEHP) causes dysbiosis in zebrafish (Adamovsky et al. 2020; Jia et al. 2021) and mice (Deng et al. 2020; Lei et al. 2019). The varying toxicity of DEHP in different rodent species was attributed to diverse microbiome compositions (Wang et al. 2020). Chronic DHEP exposure may induce obesity through disruption of host lipid metabolism and gut microbiome composition (Su et al. 2022). In humans, data are available from newborns where early-life DEHP exposure altered gut microbiome composition and diversity, specifically leading to a decrease in *Rothia *sp. and *Bifidobacterium longum* (Yang et al. 2019). For BPA, evidence for microbiome dysbiosis in zebrafish and mice is also available, and again, together with host factors (e.g., gender) an association with metabolic disorders was hypothesized (Chen et al. 2018; Diamante et al. 2021; Feng et al. 2020; Lai et al. 2016; Xu et al. 2019). In humans, data are scarce: In an in vitro model of the gut microbiome, BPA caused distinct shifts in microbial composition that were associated with hormonal effects and oxidative stress (Wang et al. 2018). In patients with binge eating disorders, BPA was elevated alongside distinct microbiome differences (Leyrolle et al. 2021). For other EDCs, e.g., nonylphenol, no data for living organisms are available yet, although environmental microbiome disruption has been described (Gálvez-Ontiveros et al. 2020; Mattana et al. 2019).

Besides alteration of the gut microbiome, it is likely that MNPs interact with the host gastrointestinal tract at a cellular level via contact with the gut epithelium. The propensity of the gastrointestinal tract to take up particles has been controversial since first discussed in The Monographs on Physiology on the “Absorption from the Intestine” by Professor F. Verzár in 1937 (Verzár, 1937). Considering the physiological conditions of the gastrointestinal tract and the pharmaceutical, biological and toxicological implications of this, systemic uptake of un-engineered particles might be considered as being of marginal likelihood (Sternson 1987) although uptake of peptides and proteins by Mucosal Associated Lymphoid Tissue (MALT) of the gut is estimated to reach 2–3% of the amount ingested and can be enhanced if the particles are “spiked” by special ligands that “boost” entry to lymphoid and non-lymphoid tissue. For example, poloxamer-coated particles have been demonstrated to be taken up at a lower level in the gastrointestinal tract of rats compared to untreated PS particles, whereas covalently bound tomato lectin and invasin molecules lead to increased systemic uptake; lectin is suspected to interact with cell surface carbohydrate moieties, and invasin mimics bacterial pathogens influencing immunogenic cell responsiveness through pathogen-associated molecular patterns (PAMPs) (Ashwood et al. 2007; Brett et al. 1993). The tomato lectin effect has been exemplified in animal experiments using PS latex particles, which showed a marked increase in systemic uptake accountable to lymphoid tissues (Hussain et al. 1997). However, after tissue uptake PS particles seem to be excreted mainly in bile, reaching levels of 18% for 50 nm, 8% for 500 nm, and 1% for 1-µm-sized PS particles (Jani et al. 1996). Another mechanism proposed, that is supportive of particle uptake into tissue, is represented by the ability of bile acids to absorb the insoluble food additive, calcium phosphate (used as an anti-caking agent) (Govers et al. 1994). Given the physico-chemical properties of calcium phosphate and bile acid, it is obvious that the hydrophilic, highly polar surface of calcium phosphate particles is reversed by the addition of bile acids resulting in hydrophobic particles. Most of the MNPs in the environment have the chemical structure of PE or PP, and are therefore hydrophobic in nature, too. A similar mechanism as described for bile acid-bound calcium phosphate particles can therefore also be applied to MNPs. Also, EDCs, especially BPA may affect gut barrier integrity, systemic inflammation, and translocation of bacterial products, as shown in rodents and patients with Crohn´s disease (Braniste et al. 2010; Feng et al. 2019; Linares et al. 2021). For other EDCs insufficient evidence is available to support or refute an effect on intestinal barrier function, indicating the need for additional research efforts.

Consequently, the complexity of the particle surface structure is an important aspect to consider for tissue uptake. As reviewed by Kihara et al., the “biological identity,” referred to as the “protein corona” (Cedervall et al. 2007a), dictates complex formation and hence adsorptive properties of nanoparticles (Di Silvio et al. 2017; Fleischer and Payne 2014; Lesniak et al. 2012). Proteins residing in the gut fluid can be competitively adsorbed to the particle surface with altered affinity (Cedervall et al. 2007b; Kasche et al. 2003; Tenzer et al. 2013); if bound and participating in corona formation, these proteins undergo complex structural changes with physico-chemical consequences that influence tissue uptake and complicate further investigation (Caillou et al. 2008). The role of the changing composition of environmental or biomolecular corona covering the MNP particle, from the outside to the inside of the human body, across tissue barriers, and its effects on uptake, fate, and toxicity, is understudied and deserves special attention (Vethaak and Legler 2021).

The process of particle uptake in MALT of the gastrointestinal tract has been proposed to be executed by three main routes: (1) phagocytosis, (2) transcellular uptake—via intestinal enterocytes, or (3) paracellular uptake—via tight junctions between enterocytes (Florence 1997). Accordingly, the following factors supporting particle uptake by MALT in the gut have been described: particle stability, particle diameter (< 5 µm), lack of surface charge, surface hydrophobicity, and the presence of specific ligands (Florence 1997). Of these factors, particle size is the most studied and has been determined as being important for endocytosis (< 0.5 µm) and also for phagocytosis, the latter conducted in particular by macrophages (> 0.5 µm) (Yoo et al. 2011). Phagocytosis is dependent on macrophage volume as demonstrated after intraperitoneal injection of polymethacrylate and PS particles (1, 5, and 12 µm) into mice (Tomazic-Jezic et al. 2001). Considering the sizes of the particles under consideration, and the presence of an intact epithelial barrier, transcellular and paracellular transport could be excluded as routes of particle uptake (Alberts et al. 2002). Due to increased intestinal barrier permeability, patients with inflammatory bowel disease, show an increase in MNP particle uptake of 25% compared to healthy controls after administration of engineered particles as oral drug delivery vehicles (Schmidt et al. 2013). Generally, particles with a size range of 0.1–150 µm can be taken up via the intestinal barrier by engulfment through the plasma membrane of microfold (M) cells in Peyer’s Patches (Galloway 2015; Hussain et al. 2001). According to these data, plastic particles (< 150 µm) and probably all nanosized particles are able to invade the mucosal barrier and form local deposits that might translocate to cause systemic exposure with yet unknown consequences. In evidence, the systemic exposure rate of MPs (< 150 µm) was shown to be limited (≤ 0.3%) whereby only particle fraction of < 1.5 µm penetrate deeper into organs (EFSA 2016). In contrast to larger particles (> 10 µm) where methods of detection and quantification are largely established (Cole et al. 2011; Prata et al. 2019), the identification of smaller particles is far more challenging (Correia and Loeschner 2018; Zhou et al. 2019), since uptake does not follow strict characteristic features like particle size or composition. Consequently, data on systemic bioaccumulation in distant organs are contradictory and partly inconsistent (Deng et al. 2017; Stock et al. 2019). Several studies investigating in vitro intestinal absorption were based on the widely established PS particle model; here, the uptake of PS particles (50–100 nm) varied excessively with rates ranging from 1.5 to 10% reflecting the broad physico-chemical properties of NPs (des Rieux et al. 2007; Kulkarni and Feng 2013; Walczak et al. 2015). In addition to being restricted by particle size, surface chemistry and the model system used, research might further be limited by particle degradation through chemical pretreatment resulting in an underestimation of the quantification of particles (Silva et al. 2018). A study that used Fourier-Transform Infrared (FT-IR) microspectroscopy as the method of choice for MP particle detection in human stool samples reported further detection issues with regard to the percentage cut-off set for spectral similarity of MPs with reference MPs, as well as the analytic differentiation between MPs and solids remaining after sample preparation; though various sorts of MPs (50 to 500 µm) were detected in the analyzed stool samples, no conclusions could be drawn regarding the origin or fate of MPs (Schwabl et al. 2019). While animal experiments showed that NP and small MP particles (0.5 to 5 µm) were spared from further processing and degradation due to their structural stability (Florence 1997; Smith et al. 1995), another study demonstrated the intracellular bio-persistence and long-term stability of NPs taken up by endolysosomes after passing through the modeled intestinal barrier of Caco-2 cells (Magrì et al. 2018).

## Are Complexes of Micromolecules and Plastic Particles Drivers of Carcinogenesis?

While it is clear that plastic particles can affect human health, little data are available on their role in the pathogenesis of gastrointestinal cancer which might be predicted given that the gastrointestinal tract is a major route of exposure. So far, we know that NPs migrate to organs through lymphatic and/or vascular invasion more frequently than do larger particles (Ai et al. 2011); if smaller than 100 nm, particles can hijack intestinal uptake routes to overcome physiological barriers (Pietroiusti et al. 2013), depending on their physico-chemical properties such as shape, size, material, and surface characteristics (Qiu et al. 2018). Plastics in the nanoparticle range have been associated with biochemical events crucially involved in carcinogenesis, such as genomic alterations including those that alter gene expression, and potentially affect post-translational modification (Hollóczki and Gehrke 2019; Qu et al. 2019; Zhang et al. 2020), oxidative stress (Chen et al. 2017; Cortés et al. 2020), membrane damage and DNA fragmentation (Sendra et al. 2019) as well as cytotoxicity (Gopinath et al. 2019), most of which have been described by Hanahan and Weinberg in their essay “Hallmarks of Cancer” as malignancy enabling properties (Hanahan and Weinberg 2011).

With respect to gastrointestinal cancer, a multi-endpoint toxicological study demonstrated increased uptake and intracellular accumulation of MP and NP particles in colorectal cancer (CRC) cell lines (Hesler et al. 2019). Furthermore, the administration of high PE concentrations disrupted the microbiome and induced intestinal inflammation in mice, as shown by increased IL-1 $\mathrm{\alpha }$ secretion and decreased intestinal infiltration by Th17 and Treg cells (Li et al. 2020a). Another study tested the size-dependent effects of PS particles (0.1 µm and 5 µm), but found minimal effects on cell viability, oxidative stress as well as cell membrane integrity and fluidity of CRC cell lines. However, PS particles were associated with inhibition of cell membrane transporter activity and increased production of reactive oxygen species (ROS) following arsenic exposure. ROS generation is widely known for its crucial role in the growth and proliferation of cancer cells through disturbances in cellular signaling due to their mutagenic activity (Manda et al. 2015; Poillet-Perez et al. 2015; Tang et al. 2011). According to these data, plastic particles might act as substrates for membrane transport activity and as a chemosensitizer of toxic substances (the so-called “Trojan Horse effect” (Wu et al. 2019)) and in doing so, might “boost” their carcinogenic effects.

As for the particle surface, the formation of charge-specific macromolecular complexes has been shown to affect particle uptake (Walczak et al. 2015). In addition to the above-mentioned tomato lectin-invasin complex, conjugated lipopolysaccharide (LPS), a molecule found on the outer layer of gram-negative bacteria, a pathogen-associated molecular pattern (PAMP) recognized by host cells, can boost particle uptake. LPS is recognized by toll-like receptor 4 (TLR 4) that activates several signaling pathways involved in tumor progression and is almost ubiquitously expressed by human intestinal cells (Vaure and Liu 2014). In addition to TLR signaling, LPS has been shown to activate the NF-κB pathway that induces TNFα-mediated inflammatory CRC growth (Luo et al. 2004). Indeed, Wu et al. showed that PS-coated MP beads influence NF-κB and MAP kinase pathways, cytokine–cytokine receptor interactions and TLR-induced signaling; these findings were supported by the identification of a transcriptional program reflective of increased expression of inflammatory and proliferation-associated genes in PS particle-exposed CRC cell lines. In addition, this group demonstrated that cell viability decreased when the cells were exposed to higher doses of PS particles (12.5 mg L^−1^ or 50.0 mg L^−1^ for 24 h) (Wu et al. 2020).

Recent evidence of EDC-induced alterations in fetal epigenetic programming led to invitations to produce health policies to protect humans from plasticizers including BPA, phthalates, and nonylphenols; the structural analogy to sexual hormones allows these compounds to drive or inhibit hormonal actions at a multifactorial level (Martínez-Ibarra et al. 2021; Noorimotlagh et al. 2020).

BPA, which enters the gastrointestinal tract through release from the lining of canned foods and beverage containers, has been studied since its generation and identification as a xenoestrogen (Dianin 1891; Dodds and Lawson 1936); because of evident in vivo tumor-promoting properties and the induced susceptibility to breast and prostate cancer (Keri et al. 2007; Seachrist et al. 2016), it has been proposed that it may be a human carcinogen. In vitro*,* BPA alters DNA methylation and gene expression through classical estrogen receptor (ER)-binding or through membrane-initiated signaling by GRP30; exemplarily, BPA has been shown to induce SCGB2A1 overexpression—a gene that is associated with proliferation and cancer stem cell survival as well as with response to chemo- and radiotherapy in colorectal cancer cells (Caiazza et al. 2015; Munakata et al. 2014). As for colorectal cancer, ER-ß is expressed in the epithelium of normal and malignant colon cells (Elbanna et al. 2012); in clinical studies, ER-ß expression was related to disease grade and stage and inversely correlated with tumor progression (Jassam et al. 2005; Rudolph et al. 2012); consequently, it has been hypothesized that BPA-driven disruption of ER-ß function results in the loss of its tumor protective function by inhibition of estrogen-induced pro-apoptotic signaling and gene expression (Bolli et al. 2010). Studies of hepatic genome alterations elucidated that BPA significantly influenced the miRNome and transcriptome of adult zebrafish; these gene signatures further associated with human orthologs involved in oxidative phosphorylation, mitochondrial dysfunction, and cell cycle (Renaud et al. 2017). In an isogenic mouse model, dose-dependent hepatic carcinogenesis (neoplastic and preneoplastic lesions) was found after perinatal BPA exposure (50 mg BPA/kg diet) in 10-month-old mice (Weinhouse et al. 2014); these findings might be explained by BPA-driven peroxisome proliferator-activated receptor (PPAR) overexpression, that resulted in aberrant fetal programming of the liver of mice (García-Arevalo et al. 2014). However, although BPA is detected systemically in approximately 90% of tested humans, with the highest amounts found in infants and children (Calafat et al. 2005, 2009; Edginton and Ritter 2009; Kuroda et al. 2003; Lee et al. 2008; Nepomnaschy et al. 2009), the exact carcinogenic mechanism of BPA in humans is currently not known. As for phthalates, alterations of lipid storage and metabolism are mainly driven by PPAR and pregnane-X (PRX) receptor signaling, that have been associated with uncontrolled hepatic cell proliferation and the induction of enzymes involved in steroid metabolism and xenobiotic dysfunction in mouse models (Hurst and Waxman 2004; Yavaşoğlu et al. 2014), respectively). In another study using the liver cancer cell line HepG2, nonylphenol (NP) was brought into context, with ER overexpression by activation of luciferase (Yoon et al. 2000); in addition, NP induced the carcinogenic signaling pathways ERK and TGF-ß in colorectal cancer cell lines, that together drove CRC development (Yang et al. 2017).

Although evidence regarding the involvement of MNPs in the pathogenesis of cancer is scarce (Sharma et al. 2020), comparisons with the aforementioned data can be used to extrapolate hypotheses and mechanisms that form the basis of future studies. The lack of knowledge about the effects of MNPs on the human organism and their contribution to disease development results in an urgent need for targeted research in the field of microplastic–health interactions. In particular, it is important to understand which disease mechanisms triggered by MNPs can lead to carcinogenesis as well as concomitant inflammatory and immunological effects. To do this, we need to address the technological means by which MNPs can be detected in human tissues.

## Analytical Insights: Detection, Quantification, and Tracking of Plastic Particles

The chemical analytical study of MP is in part well-established. The most commonly applied measurement principles are simple observations by the human eye, optical microscopy, Fourier transformation infrared (FT-IR) spectroscopy and microscopy, Raman spectroscopy and microscopy, gas chromatographic separation (GC) and mass spectrometric detection (MS) after pyrolysis (py-GC–MS), or pyrolysis–gas chromatography time of flight mass spectrometry (py-GCToF) following thermo-extraction and desorption (TED-GC–MS). Raman spectroscopy (RS) and FT-IR microscopy methods are particularly advantageous over MS methods as they require minimal sample preparation and quantities of material, and have a very high throughput. Raman spectroscopy has the additional advantage compared to FT-IR of a wider spectral coverage, better resolution and lower water interference, the latter aspect being important for in vitro and in vivo studies (Araujo et al. 2018). Taken together, analysis of MP is mainly performed using FT-IR and RS (Renner et al. 2018) and recently a fully automated MP identification method based on FT-IR was presented, which assigns > 98% of MP correctly (Renner et al. 2019).

Analysis of tissues in the process of histopathological diagnostics is largely conducted with formalin-fixed paraffin-embedded (FFPE) tissue. It has recently been shown that the detection of MP that have incorporated into tissues, should also be possible within FFPE material. This would allow correlations to be made between the amount and type of MP and their potential association with the severity and progression of diseases. This was recently demonstrated by the detection of silicon particles in histopathological slides from women with leaking breast implants using stimulated Raman scattering imaging (van Haasterecht et al. 2020). This technology will also help to specify the role of PS MNPs in cancer formation (Gonçalves et al. 2018). In order to analyze and correlate the increase in plastic production from the early 1950s to today, with an increase in the concentration of MP in a large number of FFPE tumor samples and with clinical endpoints, it will be necessary to expand and automate this technology to develop a high-throughput approach.

NPs (i.e., < 0.1 µm) are more difficult to detect and quantify. The detection of smaller particles by the commonly applied RS and FT-IR microscopy techniques is limited by the diffraction limit of the optical microscope, which is about 0.3 µm. Hence, there is the potential for single particle analysis. However, the smaller the particles and therefore the resolution requirements of the instruments are, the longer the screening of larger tissue areas will take. This limitation is most obvious with the latest developments in instrumentation combining atomic force microscopy (AFM) with FT-IR (AFM-IR) or RS (Tip Enhanced Raman Spectroscopy, TERS), allowing the study of particles at a lateral resolution of 10 nm (Schwaferts et al. 2019). TERS also enables the investigation of a material’s properties, but experimental difficulties and the sensitive equipment make routine measurements more difficult. Thus, for the study of MNPs, tissue screening methods for localizing MNP particles in neoplasia of large areas require sophisticated instrumentation e.g., IR-AFM or detailed material identification. To our knowledge, this combination for the systematic study of MNPs in malignant tissue is not applied routinely and will require further methodological development including in situ identification with a focus on sample preparation. However, the challenge remains to track MP and possibly NP with respect to adsorption, distribution, and excretion, non-invasively, in animals and humans. A major challenge for this, will be to sample and analyze, as far as possible, in plastic-free settings, for example by avoiding contact of body tissues and fluids with plastic medical devices and using clean air chambers.

Several studies have applied carbon-14 to the investigation of MNP accumulation and distribution in seafood (Al-Sid-Cheikh et al. 2018). For relatively small animals, like scallops, the ingestion and distribution of carbon-14 labeled microplastics can be followed by measuring emitted radiation. Carbon-14 can be used in humans during ADME studies, but its application to MNP detection is limited. The regulations for carbon-14 studies only allow a single micro-dose (3.7 MBq), followed by the radioanalytical determination of excreta and plasma (Beaumont et al. 2014). Real-time biodistribution of carbon-14 labeled MNPs is not possible due to the low energy released during its relatively long half-life and a short travel distance for the beta-radiation of carbon-14 in tissue. Present-day medicine offers contemporary hybrid imaging modalities that open up the possibility of tracking such MNP in real time (2017). The imaging modality of choice has to fulfill defined requirements covering (a) high sensitivity to detect very low amounts of MNPs in a complex matrix, (b) high resolution to precisely locate the position of enrichment of the MNPs, and (c) non-invasiveness to allow long-term studies.

Live imaging modalities that satisfy these requirements include positron-emission tomography and single-photon emission computed tomography (SPECT), both applied as hybrid techniques coupled to computer tomography (CT), magnetic resonance tomography (MR), or dynamic near-infrared fluorescence (DNIF) imaging, which is still under development for whole-body coverage in humans (Huang and Pu 2020; Piper et al. 2013). Both techniques require the introduction of a label, either a radio- or fluorescence label for a detectable and verifiable signal. Beyond that, the label has to be non-toxic, metabolically stable and the chemical structure of the applied MNPs should not be changed to avoid any alterations in their biochemical behavior. Not all MNPs have the chemical properties needed to introduce such a label. In particular, PE and PP particles do not have the required functional groups for direct labeling of the degraded plastic particle (see chemical structures in Table 1). Here, the label has to be chemically introduced during the production of the plastic, before the degradation process takes place, and the respective break down into smaller pieces to yield MNPs has to be simulated. Considering the demands for both, the imaging technology and the signal delivering label, it is obvious that the mentioned imaging methodologies have their advantages, but also significant limitations.

Nuclear medicine-based techniques including positron-emission tomography (PET) and SPECT require positron or photon-emitting radionuclides to detect traceable MNPs. The ability to use the PET radionuclides carbon-11 (half-life 20 min) and fluorine-18 (half-life 110 min) allows radiolabeling with a barely altered chemical structure (authentic labeling) and subsequently high metabolic stability and very high sensitivity of detection. The major limitation of the very short synthesis and imaging time restricted by the half-life of the radionuclide remains, limiting the real-time tracking of the MNPs of up to just one day. SPECT radionuclides, like iodine-123 (half-life 13.22 h), significantly expand the time-frame of tracking MNPs, but it goes hand in hand with a loss of spatial resolution compared to PET (Khalil et al. 2011).

DNIF will be highly suitable for tracking MNPs in animals but not in humans, as this technique has not been implemented in routine clinical practices so far. DNIF permits deep-tissue imaging of fluorescently labeled plastic particles with high signal sensitivity and specificity. However, the introduction of fluorescence entities on to MNPs will significantly alter their chemical composition and presumably, their biochemical behavior too. An advantage compared to PET and SPECT are the resolved time constraints enabling long-term studies.

## Ex Machina: Molecular Modeling to Understand the Adverse Effects of NPs on Biomolecular Systems

The immense array of conceivable MNPs poses a significant challenge for research. Using realistic mixtures—e.g., derived from environmental samples—would make the identification of structures and the delineation of effects difficult, while simpler samples may overlook whole families of less prevalent but more toxic compounds, as well as synergistic or combinatorial effects. To consider all these possibilities in a rational manner is impossible, and probably also unnecessary.

In the past few decades, molecular modeling has become an invaluable tool to overcome such obstacles and to improve our understanding of the principles that govern complex biological systems at the molecular level (van Gunsteren et al. 1997; Warshel 1997). In silico studies have proven extremely useful to define new directions for research, and increase efficiency through reducing the number of costly unsuccessful experiments (van Gunsteren and Berendsen 1990). Exploiting the predictive character of theory and modeling regarding the behavior of materials have aided screening for molecular properties of interest in large libraries of compounds, e.g., in drug design (Congreve et al. 2005). Similarly, modeling could provide the kind of structural and systematic understanding which may facilitate an exploration of the highly complex and important field of NPs.

In the last decade there have been several successful attempts to acquire practical, useful information regarding NP–biomolecule interactions from modeling. It has been observed in in silico experiments that for NPs, entering the hydrophobic core of lipid bilayers is energetically favorable and in a certain size range, this process may occur without a free energy barrier (Thake et al. 2013). PP and PS particles spontaneously disentangle in lipid membranes, while PE chains remain aggregated (Bochicchio et al. 2017; Hollóczki and Gehrke 2020) (Hollóczki and Gehrke 2019; Rossi and Monticelli 2014). The structure and dynamics of the membrane are altered in a manner that suggests physiological effects (Rossi et al. 2014). Upon contact with NPs, the propensity of a model protein to form α-helical and β-loop secondary structures was shifted, with the changes being qualitatively different for PE and nylon 6,6 (Hollóczki and Gehrke 2019). Furthermore, the behavior of PE-NP in various solvents was explored through molecular modeling, revealing that in micro-structured amphiphilic liquids, e.g., ionic liquids, control over the disintegration (i.e., disentanglement) of NP analytes into free polymer chains can be achieved, which could be applied to extraction strategies for analytical purposes (Elfgen et al. 2020).

The wide spectrum of these data shows how multifarious and fruitful modeling can be when applied to problems related to NPs. On the other hand, for a better alignment with experiments, it will be extremely important in the future to move toward a higher level of complexity, and to incorporate certain realistic aspects of NPs into the applied models. This includes not only larger particle sizes, but also the introduction, for instance, of protein and environmental coronae, as well as oxidized groups when investigating the biochemical effects of these materials, as their surface structure will define most of their impact on their environment (Kihara et al. 2021).

## Outlook: Facing the Possible Consequences of MNPs on Human Health

Considering the above, there is evidence, that pollution through MNPs represents a health risk. It could be a health risk that may be irreversible, and the more plastic that is produced, the more the next generation will have to suffer its effects, which are not yet fully understood. Despite all of this evidence, plastics will remain an irreplaceable part of daily life for the time being. On the one hand, in the field of communication technology and healthcare, both of which are strongly linked to the social aspect of sustainable development. On the other hand, technologies that promote resource-efficient lifestyles with environmental sustainability rely on plastics and their versatile properties and potentials. The environmental impact of plastics is largely determined by product eco-design, retailer responsibility, and the efficiency of waste management systems. Experts and stakeholders from different disciplines need to work together to solve the complex interrelationships between plastic waste and the formation of MNPs. The polymer industry itself is called upon to provide biodegradable plastic products or safe-by-design products. With the increasing production of plastics, there is also a growing need to finally investigate the interaction of MNPs with living organisms both systematically and comprehensively, and their possible connection with the pathogenesis of disease.

**Fig. 1:**
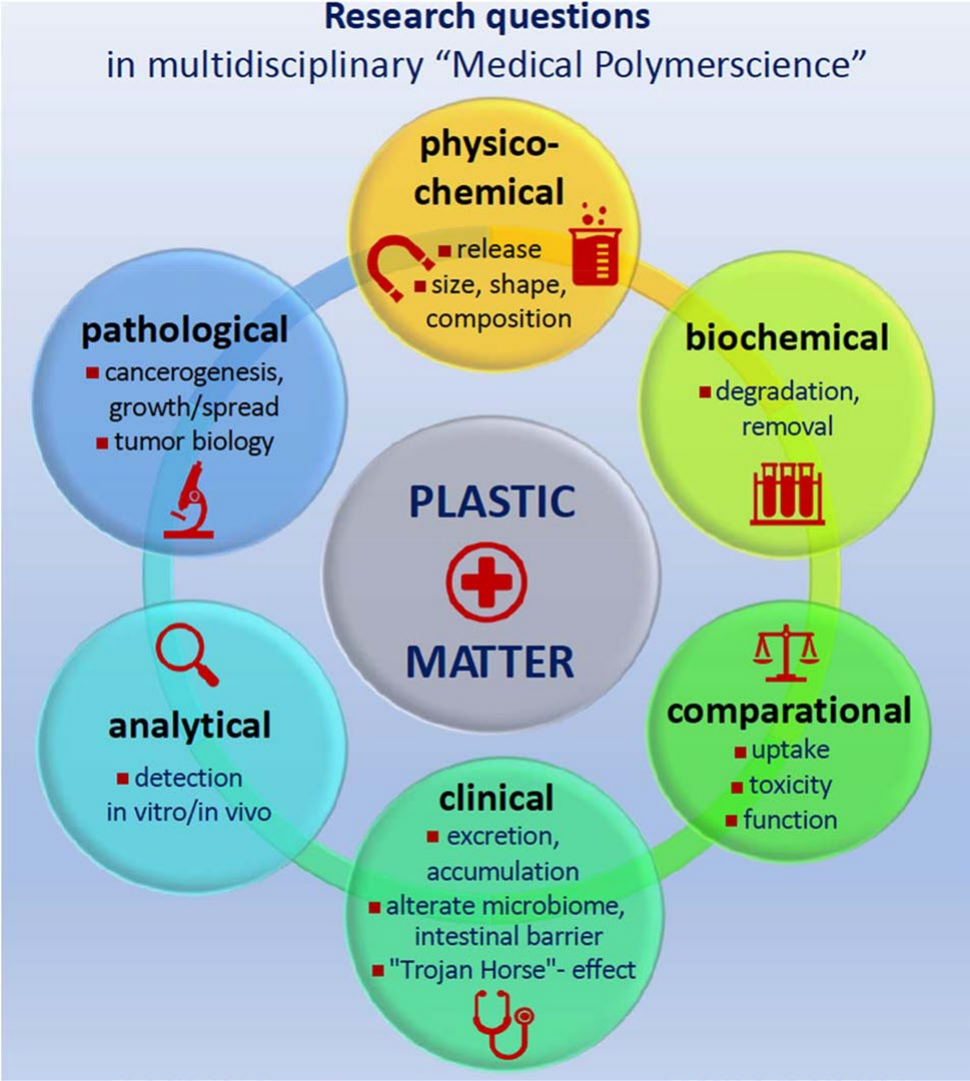
Research questions in consistence with the Sustainable Development Goals of the United Nations

What the media back in 1950 praised as the “matter plastic” has now turned into a “plastic matter.” The widely understudied interaction of MNPs and human health represents a major issue on many fronts of specialized disciplines. Now, representatives from dedicated disciplines are called to join forces to build on the above discussed fundamentals within the scope of a recently emerging research field termed “medical polymer science.” In accordance with the Sustainability Development Goals of the United Nations, the following prominent research questions must be defined (Fig. 1):

1. Physico-chemical matter: What quantities of MNPs are released from food and beverage packaging, synthetic clothes, or other plastic items? What is the size, shape, and composition of these particles?
2. Clinical matter: What fractions of ingested MNPs are excreted? Have excreted particles undergone degradation during gastrointestinal transit? Do these particles change the human microbiome composition or alter the intestinal barrier? Do they accumulate in the gut or in distant tissues and to what extent? Are there any local influencing factors such as dysbalanced microbiota or increased permeability that boost MNP tissue uptake? Is the “Trojan Horse effect” divertible to therapeutic substances; to induce local cancer treatment by targeting particle-driven signaling pathways?
3. Analytical matter: How can we detect MNPs in situ*,* in cell culture systems and/or tissue samples? How can we track MNPs on their way through the body?
4. Pathological matter: If accumulated in gut tissue, do MNPs and associated contaminants contribute to cancer development, growth and spread to other organs? Does a high MNP concentration in cancer cells correlate with high-grade tumors with poor differentiation states and poor prognostic clinical endpoints?
5. Biochemical matter: Are there potential biochemical mechanisms to remove or degrade MNPs resident in gut tissue?
6. Comparison matter: Do MNPs exhibit different uptake and toxicity profiles and pathological signatures compared to other absorbed anthropogenic or natural particles such as soot, aluminosilicates or artificial TiO_2_ nanoparticles?

## Conclusion

The above-mentioned research questions in “Medical Polymerscience” will help us to understand MNPs for their ability to interact with other substances and materials, which challenge the “plasticity” of the human body. The philosopher Catherine Malabou differentiates “creative plasticity” from “pathological plasticity,” which is based on phenomenological analysis of the human brain (Malabou 2008), that further applies to the whole human body. Creative plasticity affects all life and takes place as a balance of destruction and construction. Pathological plasticity highlights that this balance cannot be kept, it changes the human being in a primarily destructive way. In relation to plastic, especially to MNPs, it remains to be answered how we can avoid “pathological plasticity” on a physical level. To what extent plastic production introduces MNPs into the world that changes the human on a biological level in such a way that we are confronted with new or known, but modified diseases. Our aim is to understand “intra-action” of MNPs with the human body at the organ level, at the cellular level and at the protein level using the methods detailed above to find more sensitive analytical methods that will detect MNPs in vivo*,* or try to understand their ways of “intra-action” using new in silico studies. More detailed research on how MNPs affect the structures and processes of the human body, and whether and how MNPs can transform cells and induce carcinogenesis is urgently needed, particularly in light of the exponential increase in plastic production and the ensuing accumulation of non-degradable MNPs, the problem is becoming more urgent with each day.

## Data Availability

Enquiries about data availability should be directed to the authors.
